# Novel *Antrodia cinnamomea* Extract Reduced Cancer Stem-Like Phenotype Changes and Resensitized *KRAS*-Mutant Colorectal Cancer via a MicroRNA-27a Pathway

**DOI:** 10.3390/cancers11111657

**Published:** 2019-10-26

**Authors:** Tsung-Jen Lin, Kuo-Chu Lai, An-Sheng Lee, Chien-Hsin Chang, Chiung-Lin Liu, Ching-Hu Chung

**Affiliations:** 1 Program in Pharmacology and Toxicology, Department of Medicine, School of Medicine, Tzu Chi University, Hualien 97004, Taiwan; 104752101@gms.tcu.edu.tw; 2 Cardiovascular Research Center, Buddhist Tzu Chi General Hospital, Hualien 97004, Taiwan; 3 Department of Pharmacology, School of Medicine, Tzu Chi University, Hualien 97004, Taiwan; kuochu@gms.tcu.edu.tw; 4 Department of Medicine, Mackay Medical College, New Taipei City 25245, Taiwan; anshenglee33@gmail.com; 5 Institute of Pharmacology, College of Medicine, National Taiwan University, Taipei 10051, Taiwan; amy.mit@yahoo.com.tw; 6 Oneness Biotech Company, Ltd., Taipei 10680, Taiwan; agg0110@gmail.com

**Keywords:** colorectal cancer, *Antrodia cinnamomea*, *KRAS*, resensitization, miRNA-27a

## Abstract

Colorectal cancer (CRC) is one of the most common causes of death in Taiwan. Previous studies showed that *Antrodia cinnamomea* (AC) can treat poisoning, diarrhea, and various types of cancer. Therefore, we purified a novel ubiquinone derivative, AC009, and investigated its antitumor effects. Cell viability assays revealed that AC009 reduced the viability of several human CRC cell lines. AC009 treatment resulted in cell-cycle arrest/apoptosis, and these effects may occur via caspase and Bcl-2 signaling pathways. We demonstrated that AC009 could significantly inhibit in vivo tumor growth in xenograft mouse models. Using messenger RNA (mRNA) and microRNA (miRNA) microarrays, we found that *KRAS* gene expression was also regulated by AC009, possibly through specific miRNAs. AC009 also reduced cancer stem-cell marker CD44^+^/CD24^+^ expression and restored the tumor inhibition effect of cetuximab in *KRAS*-mutant CRC. Moreover, we found that miRNA-27a could restore the tumor inhibition effect of cetuximab in *KRAS*-mutant CRC cells. Taken together, our results suggest that AC009 has therapeutic potential against human wild-type and *KRAS*-mutant CRC.

## 1. Introduction

Cancer is the leading cause of death in Taiwan, and colorectal cancer (CRC) ranks third in cancer mortality. More than 135,000 new cases of CRC are diagnosed annually in the United States of America (USA) [1]. In CRC, cancer cells and tumors grow in the colon or rectum. Diet, smoking, alcoholism, and obesity are the main causes of CRC [2,3,4]. In addition, previous research indicated that a lack of water uptake is an important factor in the development of cancerous cells and tumors [5]. The worldwide incidence of CRC varies widely from country to country [6]. This may be attributed to differences in diet, environmental exposure, and genetics.

According to statistics from the USA, CRC resulted in approximately 715,000 deaths in 2010, up from 490,000 in 1990 [3,7]. In early-stage CRC patients, there are no significant symptoms. Most CRC patients are diagnosed after a health check-up or screening. The increase in CRC screening led to more cases being diagnosed at an earlier stage and more effective treatments that improve outcomes in developed countries. Although CRC mortality decreased since the mid-1980s, metastatic CRC is still associated with high mortality rates [8].

Oncogene expression is involved in modulating radiotherapy and chemotherapy responses due to its effects of proliferation, angiogenesis, and inflammation. *KRAS* is an operative oncogene that was found to be particularly important in tumor progression and treatment [9]. *KRAS* mutations promote GTPase activity and increase cellular proliferation and transformation to malignant cells [10]. KRAS protein is activated by extracellular signals, such as growth factors, and the epidermal growth factor receptor (EGFR) is the most important target in colorectal carcinogenesis [11]. Cetuximab is an EGFR-blocking monoclonal antibody, and it was approved for the treatment of metastatic CRC. *KRAS* mutations are poor prognosis biomarkers and negative predictors for cetuximab efficacy [12]. Because anti-EGFR therapy is the most efficacious in patients with wild-type *KRAS* metastatic CRC, suitable treatment for *KRAS*-mutant metastatic CRC still requires further investigation.

*Antrodia cinnamomea* (AC) is a fungus that grows on the rotten wood of camphor trees and belongs to the family Polyporaceae. In traditional herbal medicine, AC is used to treat food poisoning, poisoning, hypertension, and liver cancer. Most studies of AC confirmed its wide range of biological activities, including antitumor, antioxidation, antihypertension, cholesterol-lowering, and anti-inflammatory properties [13]. In previous studies, the ubiquinone derivative 4AAQB, which is extracted from AC, showed excellent antitumor potential in HepG2 liver cancer cells [14]. Moreover, in other cancer cells, such as breast cancer and prostate cancer cells, AC showed significant cell viability inhibition effects [15,16].

A fermented culture broth of AC was reported to inhibit cancer cell growth and migration via mitogen-activated protein kinase (MAPK) pathway regulation [13]. Another extract from AC, YMGKI-1 (3-[4-(3-methylbut-2-enyloxy)phenyl]-4-isobutyl-*N*-hydroxypyrrole-2,5-dione), was found to promote autophagy in head and neck cancer stem cells and activate E-cadherin to further increase differentiation [17]. Moreover, Bcl-2 family genes play an important role in cancer cell apoptosis. It was found that herbal medicine treatments can cause Bcl-2 and Bcl-x(L) downregulation [18]. However, the detailed mechanisms of ubiquinone derivatives for CRC treatment are not clear. In this study, we found that another ubiquinone derivative (AC009) is involved in the regulation of caspase and Bcl-2 signaling. The oncogene *KRAS* and cancer stem-cell (CSC) marker CD44^+^/CD24^+^ expressions were also suppressed by AC009. Cetuximab-resistant *KRAS*-mutant CRC cells were resensitized upon AC009 treatment, and this effect may have been mediated by microRNA (miRNA)-27a. Our results suggest that AC009 may have therapeutic potential against *KRAS*-mutant CRC.

## 2. Results

### 2.1. Effects of AC009 on CRC Cell Proliferation

**Figure 1:**
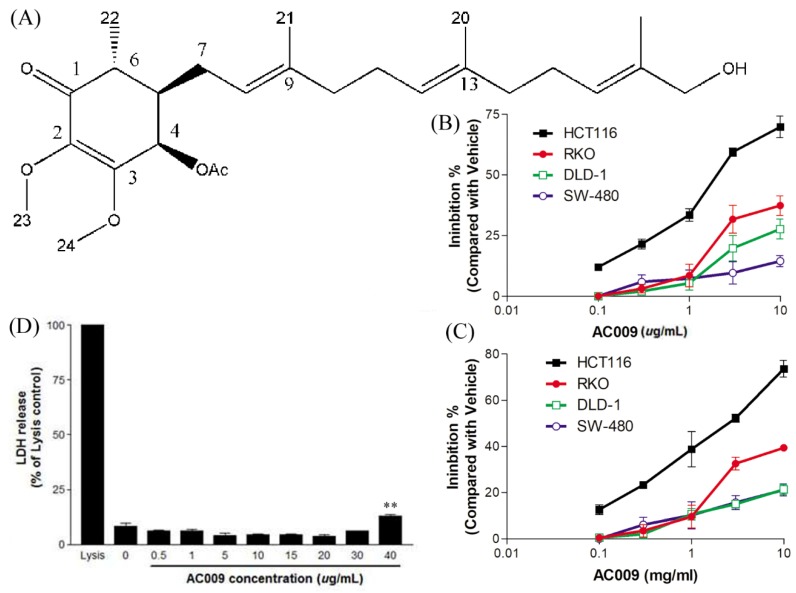
The structure of AC009 and its effect on colon cancer cells viability. The structure of AC009 is shown in (**A**). Colorectal cancer (CRC) cells (HCT116, RKO, DLD-1, and SW-480) were starved for 48 h, cultured with DMEM (with 10% FBS) in the presence or absence AC009 (0.1, 0.3, 1, 3, 10 μg/mL). The cell viability was detected by sulforhodamine B (SRB) assay (**B**) and crystal violet assay (**C**) after 72 h; the results are presented as percentages of inhibition (*n* = 10). (**D**) The cytotoxicity of colorectal cancer cells treated with different concentrations of AC009 (HCT116 cells, 72 h treatment; *n* = 10, mean ± SEM, ** *p* < 0.01).

The structure of AC009 is shown in Figure 1A. Four CRC cell lines (HCT116, RKO, DLD-1, and SW480) were used to examine the inhibitory effects of AC009 after 72 h of treatment using sulforhodamine B (SRB) cell survival and crystal violet assays. The data showed that AC009 inhibited the survival of all four CRC cell lines, and the crystal violet assay results were similar to the SRB assay (Figure 1B,C). However, AC009 had the greatest dose-dependent effect in HCT116 cells. The 50% inhibitory concentration (IC_50_) of AC009 was 2.56 µg/mL. A lactate dehydrogenase (LDH) release test was used to test the cytotoxic effect of AC009. Figure 1D shows that AC009 was cytotoxic in HCT116 cells at 40 µg/mL. According to this result, AC009 inhibited HCT116 cell growth via antiproliferative effects, but its cell cytotoxicity effects were not significant.

### 2.2. Effects of AC009 on HCT116 Cell-Cycle Arrest and Apoptosis

**Figure 2:**
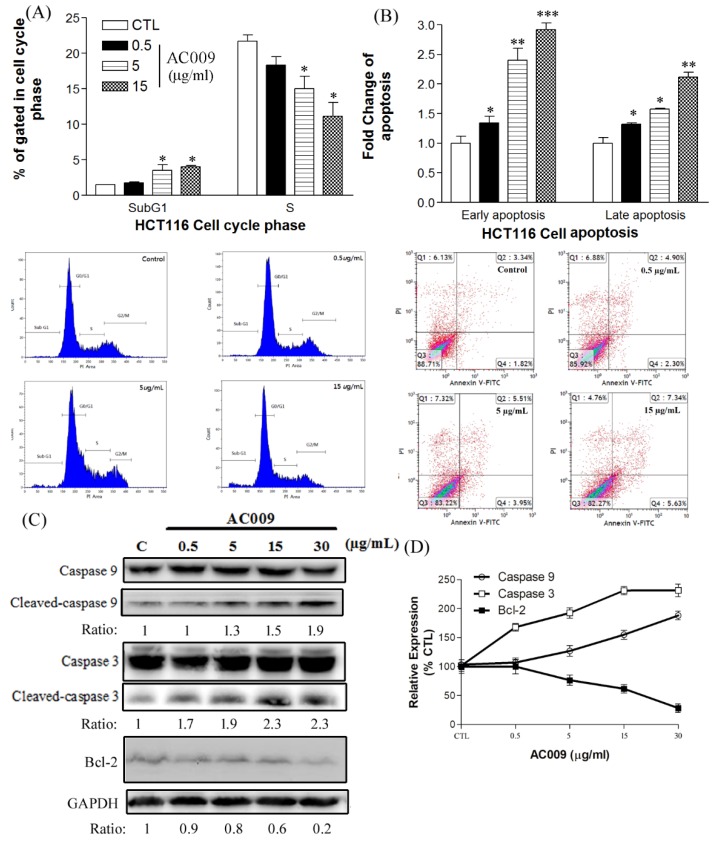
Effects of AC009 on HCT116 cell-cycle arrest, the promotion of apoptosis, and caspase/Bcl-2 expression. A flow cytometry assay was used to detect the cell cycle and annexin V apoptosis of HCT116 cancer cells after AC009 treatment. (**A**) Cell-cycle change after AC009 treatment at different concentrations for 48 h. (**B**) Annexin V-FITC shows early- and late-phase apoptosis after AC009 treatment for 24 h. The fold-change of early and late apoptosis was analyzed (*n* = 10, mean ± SEM, * *p* < 0.05, ** *p* < 0.01, *** *p* < 0.001). (**C**) In caspase, the detection results show that cleaved caspase 9 and cleaved caspase 3 increased. The Bcl-2 and GAPDH expressions were also detected (*n* = 3). The ratios of these proteins and their controls are shown below the band. Quantitative analyses of cleaved caspase 9, cleaved caspase 3, and Bcl-2 are presented as the mean density, as determined by a densitometer (**D**).

To further validate AC009 treatment-induced changes, flow cytometry was used to analyze cell-cycle changes and sub-gap 1 (G1)-phase apoptosis in HCT116 cells. Consistent with the cell viability results, a high apoptosis ratio with cells in the sub-G1 phase was induced by AC009 treatment (5 and 15 µg/mL, Figure 2A). Moreover, the number of HCT116 cells in the synthesis (S) phase decreased with AC009 treatment (5 and 15 µg/mL, Figure 2A). To further confirm the changes in early-phase and late-phase apoptosis, an annexin V-FITC assay was performed. The number of cells in early-phase and late-phase apoptosis were increased after AC009 treatment (Figure 2B). In addition to apoptosis, the cytotoxicity of AC009 was checked, as shown in Figure 1D. LDH release (necrosis marker) in AC009-treated HCT116 cells was extremely low. These results indicate that AC009 induced HCT116 cell apoptosis without necrosis.

### 2.3. Effect of AC009 on Caspase Activation and Bcl-2 Inhibition

Although AC009 induced HCT116 cell apoptosis, the signaling pathway and gene regulation involved were unclear. Our results showed that cleaved caspase 3 and caspase 9 were dose-dependently increased (Figure 2C), and Bcl-2 expression was decreased after AC009 treatment (Figure 2C). These results indicate that caspases and Bcl-2 are key targets in AC009-treated HCT116 cells.

### 2.4. In Vivo Antitumor Effect of AC009

**Figure 3:**
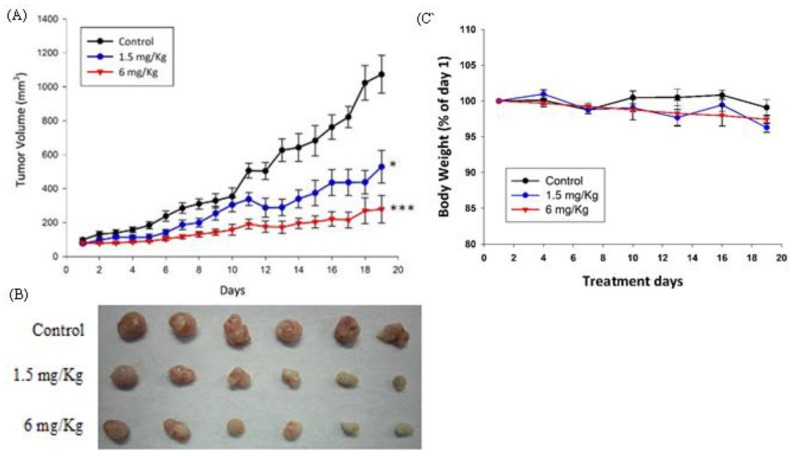
The in vivo anti-tumor effect of AC009. NOD/SCID mice were implanted with HCT116 CRC cells. Seven to 12 days later (tumor volume growth up to 2 mm^3^), AC009 was administered via intraperitoneal injection (1.5 and 6 mg/kg). (**A**) Tumor volume (mm^3^), (**B**) HCT116 picture of the control tumor, and after different concentrations of AC009 treatment. (**C**) Ratios of mice body weights (NOD/SCID mice; *n* = 10 per group, mean ± SEM, * *p* < 0.01, *** *p* < 0.001).

A xenograft animal model was used to test the in vivo effect of AC009 on tumor growth. The model was generated by the subcutaneous injection of human CRC HCT116 cells into NOD SCID mice. Once tumor growth reached 2 mm^3^, different doses of AC009 (1.5 and 6 mg/kg) were administered via intraperitoneal injection, and the tumor volume and body weight were measured every two days. AC009 at both doses significantly inhibited tumor growth compared to the control (Figure 3A). Moreover, there was no significant weight loss in the mice during AC009 treatment (Figure 3C). These results indicate that AC009 can inhibit HCT116 CRC cell growth in vivo.

### 2.5. MiRNA and mRNA Microarray Analysis

**Figure 4:**
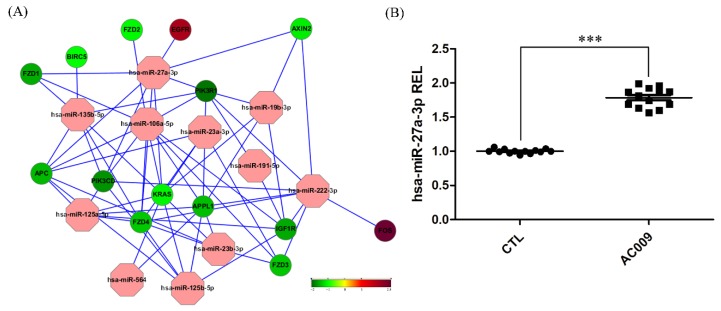
Microarray of microRNA (miRNA)/messenger RNA (mRNA) assay and qRT-PCR. (**A**) Microarray of miRNA assay analysis shows the up- and downregulation of specific genes after 24 h treatment with AC009. The green color indicates that mRNA expression decreased by 50%, and the red color indicates that mRNA expression increased 2.8-fold. (**B**) Expression levels of hsa-miR-27a-3p were normalized using the U6 small nuclear RNA (snRNA) and are expressed as REL (relative expression level); *n* = 13 per group, *** *p* < 0.001.

MicroRNA and messenger RNA (mRNA) microarrays were used to determine the specific genes or signaling pathways that were affected by AC009. As shown in Figure 4A, the oncogene *KRAS* was downregulated after AC009 treatment, and hsa-miR-27a-3p miRNA was one of the targets that affected *KRAS* expression. These results indicated that AC009 may further affect some miRNAs to influence HCT116 mutant *KRAS* gene expression. The hsa-miR-27a-3p miRNA expression in HCT116 cells was confirmed by qRT-PCR. The expression of hsa-miR-27a-3p was significantly higher in the AC009-treated group than in the normal control group (Figure 4B) (*p* < 0.001). This finding was similar to the microarray results.

### 2.6. Effect of AC009 on KRAS Protein Expression and Restoration of Cetuximab Antitumor Ability

**Figure 5:**
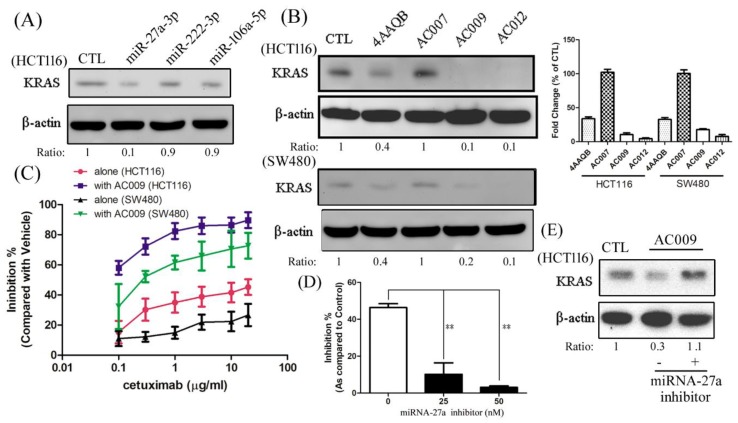
The effect of miRNA and AC009 in KRAS protein expression and resensitized cetuximab antitumor ability. After treatment with miR-27a-3p, miR-222-3p, miR-106a-5p (**A**), AC009, or its derivatives (**B**), a Western blotting assay was used to detect KRAS protein expression. HCT116 or SW480 cells were grown for 24 h, starved for 48 h, and cultured with DMEM (with 10% FBS). The cell viability (SRB assay) to detect the various concentrations of cetuximab in the absence or presence of AC009 (1 μg/mL) for the HCT116 and SW480 cells is shown in (**C**). The effect of AC009 (1 μg/mL) on HCT116 cell viability (SRB assay) in the absence or presence of an miRNA-27a-specific inhibitor is shown in (**D**). The effect of KRAS protein expression influence by an miRNA-27a-specific inhibitor and AC009 is shown in (**E**). The ratio of KRAS and β-actin is shown below the band. All data are representative of experiments performed three times. ** *p* < 0.01.

To confirm the effect of miRNA and AC009 on *KRAS* expression, several miRNAs and antroquinonol derivatives were used. After treatment with miR-27a-3p, miR-222-3p, miR-106a-5p, or various ubiquinone derivatives (1 µg/mL), *KRAS* expression was measured. KRAS was almost completely inhibited by miR-27a-3p and AC009 and was partially inhibited by 4AAQB (Figure 5A,B). We further tested the effect of AC009 in a resensitized cetuximab-resistant cell line. The IC_50_ of cetuximab alone for HCT116 growth inhibition was more than 20 µg/mL (Figure 5C). When combined with AC009 (1 µg/mL), the IC_50_ of cetuximab was reduced to less than 0.1 µg/mL (Figure 5C). The resensitization effect of AC009 was similar to SW480 cells. Additionally, an miRNA-27a-specific inhibitor was used to confirm that the antitumor effects of AC009 were mediated by miRNA-27a. The antitumor effects of AC009 were diminished after miRNA-27a-specific inhibitor treatment (Figure 5D), and KRAS protein expression was upregulated by miRNA-27a-specific inhibitor treatment (Figure 5E). These results indicate that AC009 may decrease mutant *KRAS* expression via miR-27a-3p and resensitize cells to the antitumor activity of cetuximab.

### 2.7. Effect of AC009 on Restoring Cetuximab Antitumor Ability in In Vivo Models

**Figure 6:**
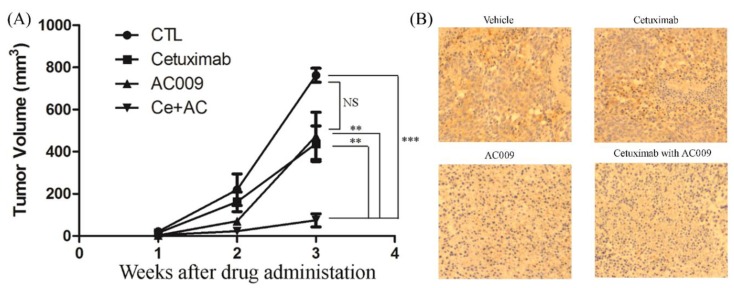
The effect of AC009 resensitizes the cetuximab anti-tumor ability in in vivo models. The tumor volume (mm^3^) of cetuximab in the absence or presence of AC009 inhibited HCT116 tumor growth in the NOD/SCID mouse xenograft (**A**). The expression of *KRAS* in a HCT116 tumor xenograft was studied (**B**). ** *p* < 0.01, *** *p* < 0.001.

Tumor-bearing mice were intraperitoneally administered cetuximab with or without AC009 at 0.1 mg/kg/day. The administration of cetuximab alone showed no significant suppression of tumor growth compared to control treatment (Figure 6A). In combination with AC009 (0.1 mg/kg), cetuximab significantly suppressed tumor growth compared to control treatment (Figure 6A). Groups treated with AC009 alone or combined with cetuximab also displayed a significant decrease in *KRAS* expression in HCT116 CRC cells (Figure 6B). These results show that AC009 decreases mutant *KRAS* expression and restores the antitumor activity of cetuximab in vivo.

### 2.8. Effect of miR-27a on Restoring Cetuximab Antitumor Ability

**Figure 7:**
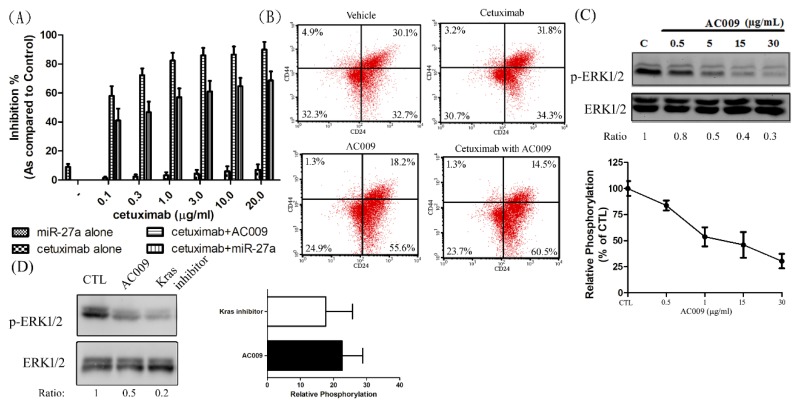
The effect of miR-27a on the resensitized cetuximab anti-tumor ability, and the effect of AC009 in cancer stem-cell marker expression. HCT116 cells were transfected with miR-27a mimics and grown for 24 h, starved for 48 h, cultured with DMEM (with 10% FBS), and treated with various concentrations of cetuximab in the absence or presence of AC009 (1 μg/mL), and cell viability was detected (SRB assay) (**A**). A flow cytometry assay was used to detect CD44 and CD24 expression on the HCT116 cell surface (**B**). After AC009 treatment (**C**) or KRAS inhibitor treatment (**D**), the ERK phosphorylation was studied by Western blotting, and the mean density was determined by a densitometer. The ratio of p-ERK1/2 and ERK1/2 is shown below the band. All data are representative of experiments performed three times.

To confirm that AC009 resensitized cells to cetuximab and that its antitumor ability was mediated by miR-27a expression upregulation, we transfected a miR-27a mimic and determined its effect on restoring cetuximab antitumor ability. After transfection with miR-27a, HCT116 cells were susceptible to cetuximab treatment, and the inhibition pattern was similar to that of cetuximab combined with AC009 (Figure 7A).

### 2.9. Effect of AC009 on Cancer Cell Stemness

According to another study, the ubiquinone derivative 4AAQB might inhibit cancer stem-cell-associated gene expression and suppress the cancer stem-cell-like phenotype [19]. We further validated the effects of AC009 on CSC marker CD44^+^/CD24^+^ expression [20]. A total of 30.1% of CD44^+^/CD24^+^ CSCs were present in the vehicle control groups, and no significant difference was observed after treatment with cetuximab (Figure 7B). In the groups treated with AC009 alone or with AC009 combined with cetuximab, the CD44^+^/CD24^+^ CSCs significantly decreased to 18.2% and 14.5%, respectively. Because ERK inhibitors may suppress the enrichment of breast cancer stem-like cell populations [21], we also determined whether the ERK pathway was involved in AC009-treated HCT116 cells. ERK activation was abolished by AC009 (Figure 7C). After KRAS inhibitor treatment in the HCT116 cell line, the tyrosine phosphorylation of ERK was also decreased (Figure 7D). These results indicate that the KRAS–ERK signaling pathway may be involved in AC009-mediated reductions in CRC stem-like phenotype expression.

## 3. Discussion

In this study, we investigated the *A. cinnamomea* extract ubiquinone derivative AC009 for CRC treatment. Firstly, we used the SRB assay to understand the antitumor effect of AC009 and crystal violet staining to confirm the results. We found that AC009 can inhibit CRC cell viability (Figure 1B) and that the HCT116 cell line yielded representative results; thus, this cell line was chosen for further study. An LDH assay was used to measure necrosis in HCT116 cells after AC009 treatment (Figure 1D). Our results revealed that AC009 induced HCT116 cell death, but not via necrosis. We further verified that AC009 induced HCT116 cell apoptosis by using flow cytometry to detect the cell cycle and sub-G1 phase ratio. We found that AC009 caused cell-cycle arrest in the sub-G1 phase (Figure 2A). This result was also confirmed by an annexin V-FITC cell apoptosis assay (Figure 2B). The number of cells in S phase also decreased after AC009 treatment (Figure 2A). Taken together, these results suggest that AC009 induces HCT116 CRC cell death via apoptosis. The Western blotting results showed that AC009 induced CRC cell apoptosis, possibly via caspase 9, caspase 3, and Bcl-2 regulation. According to these results, the effects of AC009 are most likely caspase 3/9- and Bcl-2-dependent [22]. Next, we generated a xenograft animal model using NOD/SCID mice. Consistent with the in vitro results, the xenograft animal model indicated that AC009 significantly inhibited HCT116 tumor growth in mice (Figure 3A). We also found that AC009 affects HCT116 mutant *KRAS* expression using an mRNA array (Figure 4A) and Western blotting (Figure 5B). In addition, AC009 may be further involved in KRAS downstream protein ERK activation, and this phenomenon was consistent with the results in HCT116 cells after KRAS inhibitor treatment. The induction of miRNA-27a expression by AC009 was confirmed by qRT-PCR (Figure 4B). The AC009-induced effects of *KRAS* inhibition and the restoration of the antitumor ability of cetuximab are shown in Figure 5C and Figure 6. The involvement of miRNA-27a and suppression of the cancer stem-like phenotype were also studied (Figure 7). Moreover, to verify that miRNA-27a is a key regulator of the effects of AC009 in HCT116 cells, an miRNA-27a-specific inhibitor was used. The results revealed that, after miRNA-27a inhibitor treatment in the AC009 group, the antitumor effects of AC009 were diminished and KRAS protein expression was restored (Figure 5D,E). The apoptosis induced by AC009 was not affected by miRNA-27a-specific inhibitor treatment. Thus, miRNA-27a plays a vital role in AC009 inhibition of CRC proliferation.

EGFR is a potent cancer cell growth promoter, and it is usually highly expressed on the surface of cancer cells. In addition to traditional chemotherapy, drugs that target EGFR can be used for efficient advanced colon cancer treatment [23]. Cetuximab, an EGFR-targeting antibody, proved its efficacy in the improvement of metastatic CRC patient survival [24]. *KRAS*-controlled cell growth and survival serve as a molecular switch for EGFR and the nucleus. *KRAS* mutations, as well as downstream mutations in the *BRAF* gene, may cause resistance to anti-EGFR treatment [25]. Although the importance of the therapeutic modulation of *KRAS* signaling is widely recognized, few anticancer drugs target *KRAS* [26]. In a microarray analysis, we found that *KRAS* gene expression was reduced after AC009 treatment. Although antroquinonol, which is also found in purified *Antrodia camphorata,* was reported to have RAS inhibition activity via inhibiting protein isoprenyl transferase activity in lung cancer cell lines [27], this is the first study that demonstrates that AC009 can resensitize the cetuximab-resistant HCT116 cell line and restore the antitumor effects of cetuximab. In addition, the *KRAS* inhibition effect of AC009 reduces *KRAS* expression but not kinase activity inhibition. This mechanism is not similar to that of antroquinonol. We also found that AC009 may regulate *KRAS* repression via miRNA-27a because miRNA-27a transfection had a similar resensitizing effect on cetuximab antitumor ability. miR-27a was identified as a tumor suppressor in CRC, and it may directly target *KRAS* or EGFR to inhibit tumor cell growth [28,29,30]. In metastatic tumors, miR-27a-3p mediates VE-cadherin downregulation and inhibits EMT formation to suppress tumor metastasis [31]. AC009-induced increases in miRNA-27a may occur through a similar pathway to inhibit CRC proliferation and metastasis.

## 4. Materials and Methods

### 4.1. Reagents and Chemicals

((1*R*,5*R*,6*R*)-6-((2*E*,6*E*,10*E*)-12-Hydroxy-3,7,11-trimethyldodeca-2,6,10-trien-1-yl)-2,3-dimethoxy-5-methyl-4-oxocyclohex-2-en-1-yl acetate (AC009) was produced by normal-phase column elution using a solvent gradient of hexanes/ethylene acetate from 100/0 to 70/30. The fraction of hexanes/ethylene acetate (70/30) was collected and analyzed to obtain the reference standard. DMEM (Dulbecco’s modified Eagle’s medium), FBS (fetal bovine serum), penicillin/streptomycin, and trypsin/EDTA were obtained from GIBCO (Grand Island, NY, USA). Sulforhodamine B sodium salt (SRB), trichloroacetic acid (TCA), dimethyl sulfoxide (DMSO), propidium iodide (PI), bovine serum albumin (BSA), and the miRNA-27a-3p-specific inhibitor were purchased from Sigma (St. Louis, MO, USA). Tris-base (Tris (hydroxymethyl)aminomethane) was purchased from Calbiochem. Acetic acid was purchased from Merck (Kenilworth, NJ, USA). Glycine was purchased from J. T. Baker (St, Phillipsburg, NJ, USA). The apoptosis detection kit (Annexin V FITC) was purchased from Calbiochem (Darmstadt, Germany). The Crystal Violet assay kit and lactate dehydrogenase (LDH) cytotoxicity assay kit were purchased from Bio Vision Inc. (Milpitas, CA, USA). The KRAS inhibitor (Fendiline hydrochloride) was purchased from R&D systems (Minneapolis, MN, USA). The apoptosis antibody sampler kit (caspase pathway) was purchased from Cell Signaling (Danvers, MA, USA). Antibodies specific for Bcl-2 and p-ERK1/2 were purchased from BD Transduction Laboratories (San Diego, CA, USA). Antibodies against ERK1/2 were purchased from Bioss (Woburn, MA, USA). KRAS antibody was purchased from Abcam. Beta-actin internal control antibody was purchased from Cell Signaling Technologies (Boston, MA, USA). GAPDH internal control antibody, secondary mouse IgG antibody (HRP), and rabbit IgG antibody (HRP) were purchased from Gene Tex, Inc. (Alton Pkwy Irvine, CA, USA).

### 4.2. Cell Culture

Four CRC cell lines (human), HCT116, RKO, DLD-1, and SW480, were obtained from Taipei Veterans General Hospital, and cultured in DMEM with 10% FBS and 1% penicillin/streptomycin at 37 °C, 5% CO_2_. Cells were sub-cultured every two days, and cell number changes were monitored by a hemocytometer with inverted microscopy.

### 4.3. Cell Viability Sulforhodamine (SRB) Assay 

An SRB assay was used for cell viability measurements [32]. Briefly, cells (5000 cells/96-well dish) were treated with up to 40 µg/mL AC009 for 72 h. After treatment, 50% TCA (50 µL/well) was added to each well and incubated at 4 °C for 1 h. After incubation, the supernatant was removed, washed five times with 300 µL/well ddH_2_O, and air-dried. Then, 100 µL/well 0.4% SRB solution was added to each well, and incubated at room temperature (RT) for 30 min. The supernatant was removed, washed five times with 300 µL of 1% acetic acid solution, and air-dried. After air-drying, 10 mM 100 µL Tris-base solution was added to dissolve the purple cell proteins, and absorbance was measured at 540 nm (A540). Cell viability (%) was calculated as follows:

(1) (Test sample absorbance/Control absorbance) × 100%.

### 4.4. Crystal Violet Assay

Cell growth and treatment conditions were the same as for the SRB assay. After 72 h treatment, the cell culture medium containing AC009 was removed and washed once with washing buffer gently (200 µL/well). The washing buffer was removed, and each well was stained by Crystal Violet solution (50 µL/well) at room temperature for 30 min. The supernatant was removed, washed five times with 200 µL of washing buffer, and air-dried as much as possible. After air-drying, 100 µL of solubilization solution was added to dissolve the stain, and absorbance was measured at 595 nm (A595).

### 4.5. Lactate Dehydrogenase Releasing (LDH) Assay

The assay was followed using the Bio Vision LDH cytotoxicity assay kit protocol. Briefly, HCT116 colorectal cancer cells (2 × 10^4^ cells/well) were cultured in 96-well plates overnight, and were then treated with different concentrations (background control (medium only), high control, low control, vehicle, 0.5, 1, 5, 10, 15, 20, 30, and 40 µg/mL) of AC009 for 72 h. The LDH assay kit reagent was used to detect the amount of release of LDH, and the absorbance was measured at 450 nm (A450). The LDH release concentration (%) of cytotoxicity was calculated as follows:

(2) (Test Sample − Low control) / (High control − Low control) × 100%.

### 4.6. Cell-Cycle Assay

HCT116 cells were incubated in six wells (2 × 10^5^ cells/well) with different concentrations (control, vehicle, 0.5, 5, and 15 µg/mL) of AC009 for 48 h. Then, 1× trypsin–EDTA was used to collect the HCT116 cells into 15-mL tubes, and the cells were centrifuged at 1500 rpm; the medium was removed, the cells were washed with 1× PBS, mixed thoroughly, and centrifuged at 300 rcf; then, the PBS was removed. After centrifuging, 200 µL of 1× PBS was added, and the HCT116 cells were collected to incubate overnight in ice-cold 70% alcohol solution. The next day, the cells were centrifuged at 300 rcf, washed with 1× PBS, stained with PI solution, and protected from the light for at least 30 min. After preparing the test sample, a run with flow cytometry (Beckman Gallios Flow cytometer, Beckman Coulter, Indianapolis, IN, USA) was performed to observe sub-G1 phase apoptosis and the cell-cycle changes of the cells.

### 4.7. Apoptosis Assay

The method followed the Calbiochem Annexin V FITC Apoptosis Detection Kit protocol. HCT116 cells were cultured in six wells (3 × 10^5^ cells/well) and incubated with AC009 for 24 h. Cells were collected into 15-mL tubes and centrifuged at 1500 rpm. After centrifugation, the supernatant was removed and washed with PBS. Binding buffer/annexin V-FITC solution was added, and the cells were centrifuged in a dark at room temperature for 15 min, at 1500 rpm. After removal of the supernatant, binding buffer/PI solution was added, and the mixture was centrifuged in the dark at room temperature for another 15 min. After removal of the supernatant, the test samples were run with flow cytometry (Beckman Gallios Flow cytometer) to check for early and late apoptosis.

### 4.8. Western Blotting Assay

HCT116 cells were cultured in DMEM for 24 h, and then incubated with various concentrations of AC009 or KRAS inhibitor for another 4 h. The cells were collected, applied to 10% SDS-PAGE electrophoresis, and then transferred to polyvinylidene fluoride (PVDF) membranes. Membranes were blocked by 2% BSA/0.2% PBST for 1 h at room temperature, probed with primary antibodies overnight at 4 °C, and then probed with HRP-conjugated with secondary antibodies for 1 h at room temperature. Proteins were detected using ECL chemiluminescence reagents (Millipore, Burlington, MA, USA). The molecular weights of detection proteins were as follows: caspase 3, 35 kDa; cleaved caspase 3, 19 kDa; caspase 9, 47 kDa; cleaved caspase 9, 37 kDa; Bcl-2, 26 kDa; KRAS, 21 kDa; p-ERK1/2, 42 and 44 kDa; total ERK, 42 and 44 kDa; GAPDH, 36 kDa; beta-actin, 42 kDa (Figures for whole western blot can be found in Supplementary Materials).

### 4.9. Microarray Analysis

HCT116 cells were seeded in two 100-mm^2^ dishes overnight and attached at 50% confluence. Cells were treated with or without AC009 (50% inhibition concentration (IC_50_) of AC009, 2.56 µg/mL) for 24 h, lysed with Tri-reagent, and incubated at room temperature for 15 min. Cells were scraped, removed into a 1.5-mL centrifuge tube, and analyzed by Phalanx Biotech (Hsinchu, Taiwan).

### 4.10. MiRNA Retrotranscription and Quantitative PCR

Total RNA was isolated and retrotranscribed using TaqMan^®^ MicroRNA Reverse Transcription Kit. A negative control sample was used with PBS aliquot processed as the cell sample. For individual miRNA quantitative PCR assays, samples were analyzed using miRNA-specific primers (hsa-miR-27a: Thermo Fisher^®^, catalog #4427975 and U6 snRNA: Thermo Fisher^®^, catalog #4427975, Waltham, MA, USA). Expression levels of hsa-miR-27a-3p were normalized using the U6 snRNA and were expressed as REL (relative expression level).

### 4.11. MiRNA and MiRNA Inhibitor Transfection

Briefly, the cells were cultured up to 70% confluence in the cell culture dishes. Then, 25 nM miRNA-27a-3p or a specific inhibitor solution was mixed and transfected using Lipofectamine 3000 reagent kit (Thermo Fisher Scientific, Waltham, MA, USA). After 3–5 h of incubation, 2–3 mL of complete medium was added to each culture dish and incubated continuously for 24 h. After the transfection, the cells were used for AC009 treatment, and the cell lysates were collected for further use.

### 4.12. In Vivo Xenograft Model

In this study, six-week-old male NOD/SCID mice were used, obtained from Tzu Chi University Animal Experimental Centre (Hualien, ROC). The NOD/SCID mouse is one of the best characterized models for verifying the therapeutic effect of cancer treatments for several years [33]. All of the experimental protocols regarding animal study were approved by the Laboratory Animal Use Committee of Tzu Chi University (A1050014). The suspensions with 5 × 10^6^ human CRC HCT116 cells were implanted subcutaneously into the right or left flank regions of the mice. After 7–12 days (tumor volume growth of up to 2 mm^3^), AC009 was administered via intraperitoneal injection (IP). The animals were randomized into different treatment groups (*n* = 10 in each group) and kept for 19 consecutive days. Tumor volumes were monitored by electronic caliper measurement of the length/width, calculated as follows:

(3) $$1/2\;\times \;\left(\mathrm{length}\right)\;\times \;{\left(\mathrm{width}\right)}^{2}.$$

Mice body weights were monitored every three days during the treatment. Mice were sacrificed after treatment, and the tumors were removed for the photograph. 

### 4.13. Immunohistochemistry Study

Paraffin-embedded tumors were cut into 25-μm sections, incubated with 1% H_2_O_2_, and washed three times in PBS (with Triton X-100/2% BSA). Sections were blocked with 10% BSA, and then incubated with KRAS monoclonal antibody at 4 °C overnight. After washing with PBS, sections were incubated with the biotinylated anti-mouse IgG, and incubated with 0.2% ABC solution (Vector Laboratories, Burlingame, CA, USA). After washing with PBS again, sections were treated with DAB (Sigma), mounted on gelatinized slides, air-dried overnight, and cover-slipped.

### 4.14. Statistical Analysis

Data are presented as means ± SEM. Control and experimental groups were compared by one-way ANOVA analysis and Newman–Keuls multiple comparison tests, where appropriate. A *p*-value less than 0.05 (*p* < 0.05) was considered significant.

## 5. Conclusions

CSCs are more resistant to transitional drug treatments due to their high level of drug-efflux pumps and their high capacity for DNA repair [34]. Drugs that target CSCs are needed for cancer treatment and relapse prevention. A recent study reported that other ubiquinone derivatives inhibit the expression of cancer stem-like genes [19,35]. Our data showed that AC009 also inhibited the expression of colon CSC markers (Figure 7B). The AKT and MAPK pathways are involved in CRC CSC transformation, and AKT and ERK1/2 inhibition reduces CRC cell growth [36,37]. Because AC009 inhibited ERK1/2 activation (Figure 7C), this result revealed that the ERK pathway might be involved in CRC stem-cell formation. Our data indicate that AC009 may specifically target CSCs to avoid toxicity in normal cells during cancer treatment.

**Figure 8:**
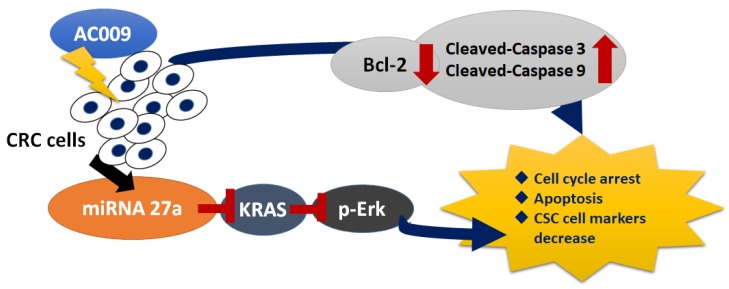
The therapeutic effects and mechanisms of AC009 in colorectal cancer (CRC) cell treatment. After *Antrodia cinnamomea* (AC) extract AC009 treatment, CRC cells may undergo apoptosis through the caspase-3- and caspase-9-dependent signaling pathways and further downregulate the Bcl-2 expression. In addition, AC009 also caused cell-cycle arrest in CRC cell treatment. AC009 also promoted miRNA-27a expression and further inhibition of *KRAS* expression. Then, p-Erk, which is a downstream protein of KRAS signaling pathway, was also downregulated and further caused the cancer stem cell (CSC) markers to decrease after AC009 treatment in CRC.

In summary, we found a novel ubiquinone derivative (AC009) with potent anticancer activity, and this is the first report that used AC009 to resensitize *KRAS*-mutant CRC cells to the antitumor ability of cetuximab. These anticancer effects were shown in both in vitro and in vivo models, and the mechanisms are shown in detail in Figure 8. We demonstrated that targeting *KRAS* is a promising strategy for treating colon cancer, especially in *KRAS*-mutant and cetuximab-resistant types. Based on our findings, AC009 will facilitate novel treatment regimens for colon cancer and could influence the design of the next generation of drugs.

## Supplementary Materials

The following are available online at https://www.mdpi.com/2072-6694/11/11/1657/s1, Figures for whole western blot.
