# Supplementary figures and images for: Novel Antrodia cinnamomea Extract Reduced Cancer Stem-Like Phenotype Changes and Resensitized KRAS-Mutant Colorectal Cancer via a MicroRNA-27a Pathway
Source: Cancers (Basel). 2019 Oct 26;11(11):1657. doi: 10.3390/cancers11111657 (PMC6896121; doi:10.3390/cancers11111657)

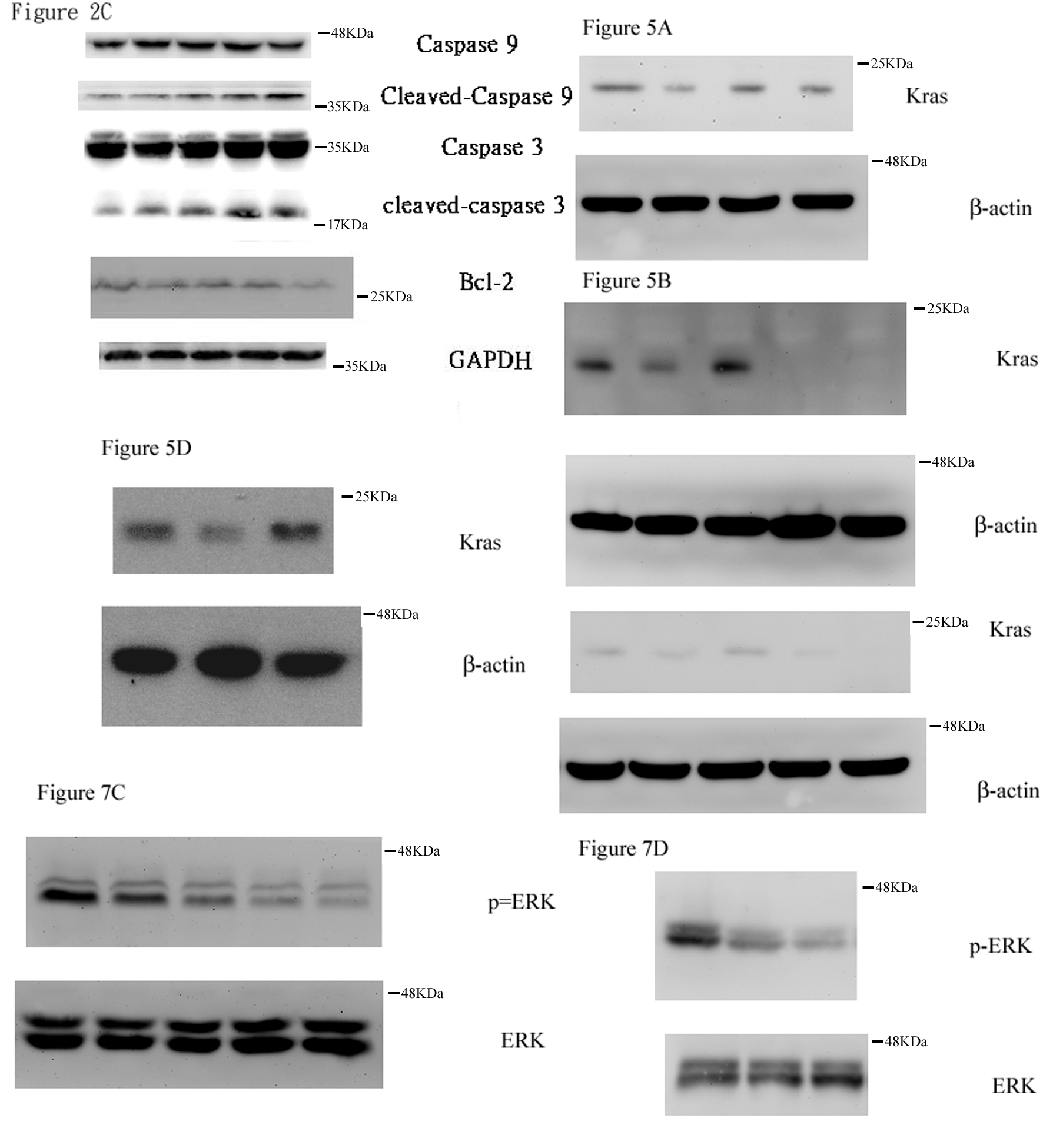

Supplement: Supplementary file 1 [file cancers-11-01657-s001.tif]
